# Three-Dimensional-Printed Poly-L-Lactic Acid Scaffolds with Different Pore Sizes Influence Periosteal Distraction Osteogenesis of a Rabbit Skull

**DOI:** 10.1155/2020/7381391

**Published:** 2020-04-23

**Authors:** Danyang Zhao, Wenbo Jiang, Yu Wang, Chuandong Wang, Xiaoling Zhang, Qingfeng Li, Dong Han

**Affiliations:** ^1^Department of Plastic and Reconstructive Surgery, Shanghai Ninth People's Hospital, Shanghai Jiao Tong University School of Medicine, No. 639, Zhizaoju Road, Shanghai 200011, China; ^2^Clinical Translational Reform and Developmental Center of 3D Printing Technology, Shanghai Ninth People's Hospital, Shanghai Jiao Tong University School of Medicine, No. 639, Zhizaoju Road, Shanghai 200011, China; ^3^Department of Cardiology, Shidong Hospital of Yangpu District, Shanghai, 999 Shiguang Road, Shanghai 200438, China; ^4^Department of Orthopedic Surgery, Xin Hua Hospital Affiliated to Shanghai Jiao Tong University School of Medicine, No. 1665, Kongjiang Road, Shanghai 200092, China

## Abstract

The repair of bone defects is a big challenge in reconstructive surgery. Periosteal distraction osteogenesis (PDO), as a promising technique used for bone regeneration, forms a space between the periosteum and bone cortex to regenerate the new bone merely by distracting the periosteum. In order to investigate the influence of distractor framework on the PDO, we utilized three-dimensional (3D) printing technology to fabricate three kinds of poly-L-lactic acid (PLLA) scaffolds with different pore sizes in this study. The *in vitro* experiments showed that the customized PLLA scaffolds had different-sized microchannels with low toxicity, good biocompatibility, and enough mechanical strength. Then, we built up an *in vivo* bioreactor under the skull periosteum of New Zealand white rabbits. The distractors with different pore sizes all could satisfy the demand of periosteal distraction in the animal experiments. After 8 weeks of consolidation period, the quality and quantity of the newly formed bone were improved with the increasing pore sizes of the distractors. Moreover, the newly formed bone also displayed an increasing degree of vascularization. In conclusion, 3D printing technology could promote the innovation of PDO devices and fabricate optimized scaffolds with appropriate pore sizes, shapes, and structures. It would help us regenerate more functional tissue-engineered bone and provide new ideas for further clinical application of the PDO technique.

## 1. Introduction

There are still a lot of disputes about the treatment of bone defects caused by trauma, tumor, infection, and congenital anomaly [1]. The repair of large bone defects, especially caused by old fractures, has been a major challenge in reconstructive surgery. With frequent traffic and industrial accidents, fracture patients have increased significantly. The function and disfigurement have brought great sufferings to these patients. In clinical practice, bone defects are usually treated by graft substitutes, guided bone regeneration (GBR), distraction osteogenesis (DO), and other techniques. As the gold standard for treating bone defects, autologous bone graft avoids the immune-related complications; however, it is limited by the origin of donors, bone resorption, osteonecrosis, and morbidity from secondary trauma surgery [2, 3]. The allogenic bone and biosynthetic materials, such as artificial bone filler, have been widely used in orthopedics and sometimes have risks of infection, immune rejection, and implant displacement because of the histocompatibility problem [4]. The GBR technology uses a layer of high molecular biological membrane as the barrier to maintain the gap for blood clots, but the volume of regenerated bone is relatively limited to cure large bone defects [5]. As for the technique of DO, the new bone is formed by gradually distracting the two separating bone blocks under the condition of corticotomy or osteotomy [6]. Although the mass of the newly formed bone is not restricted, this approach is invasive to the human body and has a long treatment cycle. It can cause fibrous ossification and nonunion in some cases [7, 8].

The PDO technique is a combination of the tissue expansion and the GBR. It builds an artificial space between the cortical bone and periosteum to produce a new bone through expanding the periosteum but with no need of corticotomy or osteotomy [9]. The periosteum has a strong ability of osteogenesis and plays an important role in the process of bone growth and fracture healing [10]. From the anatomical point of view, the periosteum is composed of two layers. The outer layer, also known as the fibrage, is formed by collagen fibers and abundant in blood vessels and nerves, which has the function of nutrition and feeling. The inner layer is made up of regular osteoblasts, which participates in the growth and proliferation of the bone [11]. As early as 1966, some scholars have found that the periosteum is rich in osteoprogenitor cells, which can be differentiated into osteoblasts with slow and stable mechanical strain [12]. Yet until 2002, Schmidt et al. were the first to histologically prove that the bone can be induced merely by distracting the periosteum, that is, the PDO [13]. In another study, Kanno et al. found that the distraction force could stimulate periosteal cells to express runt-related transcription factor 2 (RUNX2) as well as other osteogenic and angiogenic factors during bone formation [14]. These studies all confirmed that the periosteum has a strong ability of osteogenesis.

The feasibility of PDO technique also lies in another theory called the “*in vivo* bioreactor.” *In vivo* bioreactor is to create an artificial space inside the body, which takes advantages of the organisms' potential to regenerate required tissues and repair the relevant defects [9]. Many areas in the body can be used as the *in vivo* bioreactor, including muscle, fat, the subperiosteum, the subcutaneous layer, and abdominal cavity [15–18]. Stevens et al. injected hydrogels between the tibia bone and tibial periosteum of New Zealand white rabbits and obtained new callus that is identical to the native bone, which they called the artificial space bone bioreactor [18]. The technology of PDO is equivalent to construct an *in vivo* bioreactor under the periosteum, and then regenerates the new bone by the osteogenesis ability of the periosteum. Bone tissue constructed by this method is similar to autologous bone graft; it overcomes the deficiency of tissue engineering *in vitro*. Furthermore, as a layer of natural barrier, the periosteum can not only prevents the ingrowth of soft tissue but also is favorable for the filling of osteocytes [9].

After it is studied for more than one decade, the osteogenic effect of PDO was considered to be influenced by multiple factors, such as device, region, procedure, height, and cytokines [9]. Among these factors, the distraction device plays an important role in determining the final effect of new bone formation. In order to get a good result of PDO, it is indispensable to maintain a stable and continuous stretch to the periosteum. Researchers have adopted different designs and materials for their studies. Kostopoulos et al. suggested that the distraction device should be perforated to keep the communication between the periosteum and cortical bone [19]. Nowadays, 3D printing technology has attracted more and more attention; it has been already applied to the fields of oral and maxillofacial surgery, orthopedics, and tissue engineering for bone regeneration [20–23]. As a kind of additive manufacturing technology, it helps us design customized implants with accurate structure. The pore size and shape of scaffolds can be casually adjusted to meet the different requirements of biomechanical strength and cell adhesion [24]. In our previous studies, we have successfully constructed tissue-engineered bone by using 3D-printed polylactic acid (PLA)/hydroxyapatite (HA) composite scaffolds [25, 26]. Biodegradable distraction devices are in line with the idea of constructing a biomimetic bone. Poly-L-lactic acid (PLLA) is one of the safe and reliable biodegradable materials; it has been approved by the US Food and Drug Administration (FDA) and has a wide application [27–29]. In order to achieve a good effect of distracting and investigate the influence of distractor framework on the PDO, we utilized 3D printing technology to fabricate three types of PLLA scaffolds with different pore sizes. These scaffolds not only meet the requirements of distraction but also have a porous structure to guarantee the interaction between the periosteum and bone cortex. Researchers also used other advanced technologies to prepare porous PLLA materials, such as supercritical air foaming and freeze drying [30–32]. These state-of-the-art systems can adjust the porosity or composition of the material itself, but they all need some special mold processing to achieve a spatial structure. 3D printing technology could satisfy the need of personalization and directly obtain the special porous structure of any shape.

To sum up, we took advantage of 3D printing technology to fabricate PLLA distraction devices with different pore sizes and then constructed an *in vivo* bioreactor under the calvarial periosteum of rabbits by using the customized distractor to distract the periosteum. We would like to investigate the influence of pore sizes of distractor scaffolds on the effect of PDO and illustrate the feasibility and superiority of 3D printing technology. It is expected that this study could provide more novel ideas for the design of PDO scaffold and offer a more solid and reliable laboratory basis for the clinical application of the PDO technique.

## 2. Materials and Methods

### 2.1. Design of Experiment

**Figure 1:**
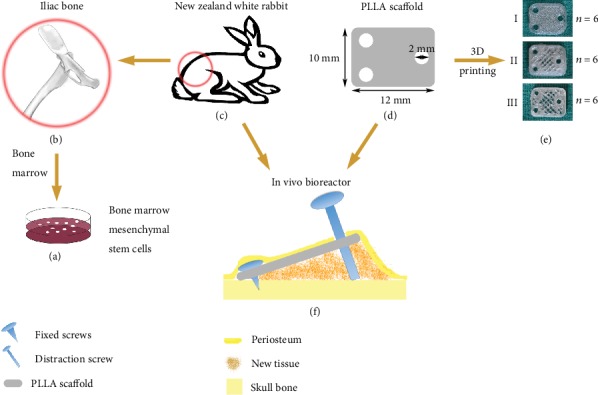
General protocol of experiment. BMSCs (a) were harvested from the iliac bone (b) of New Zealand white rabbits (c). PLLA scaffolds (12 mm × 10 mm × 1 mm, including three 2 mm diameter holes) (d) of different pore sizes (e) were produced by the 3D printer. Next, build an *in vivo* bioreactor (f) under the calvarial periosteum of New Zealand white rabbits with three different scaffolds (*n* = 6). A wedge-shaped space between the periosteum and the skull surface was formed by the customized distractor, two self-drilling titanium screws, and one self-tapping titanium screw for bone regeneration.

Eighteen male New Zealand white rabbits (6-8 weeks old, 2.5-3.0 kg) were randomly divided into three groups (groups I, II, and III, *n* = 6); each group used 3D-printed PLLA scaffolds of different pore sizes to distract the periosteum of the skull. The protocol of general experiment is shown in Figure 1.

### 2.2. Materials

Pure PLLA raw material (600000 Mw) was provided by Daigang Bioengineering Co., Ltd. (Shandong, China). Titanium screws were purchased from OsteoMed Corporation (USA). Dulbecco's modified eagle medium (DMEM)/high-glucose and penicillin-streptomycin solutions were purchased from HyClone (Logan, Utah, USA); 0.25% trypsin-EDTA and fetal bovine serum (FBS) were purchased from Invitrogen (CA, USA). All other solvents and reagents were analytical grade.

### 2.3. Design and Fabrication of PLLA Distraction Devices

The periosteum distraction device is composed of three different parts: PLLA scaffold (12 mm × 10 mm × 1 mm, including three 2 mm diameter holes), two self-drilling titanium screws (5 mm in length and 1.5 mm in diameter), and one self-tapping titanium screw (12 mm in length and 2 mm in diameter). The PLLA scaffold was fixed on the rabbit skull by two self-drilling screws at one side and then uplifted by a self-tapping screw at the opposite side to form a slope and achieve the effect of periosteum distraction.

The 3D printer (Fochif Tech, China) served in our study is based on the melt-deposition system (MDS) as our previous studies [25, 26]. Firstly, the overall structure of PLLA scaffold was accomplished by a computer-aided design (CAD) module. The designed parameters were input into a computer-aided manufacture (CAM) system to direct the motion path of the 3D printer nozzle. PLLA raw material was melted at the temperature of 180°C, and a nozzle with a diameter of 0.35 mm was selected to print at a printing speed of 60-80 mm/s. The melting PLLA material was dispensed through the piston and syringe system to produce the multilayered construction. After trimming the excess material, the mass production of PLLA scaffolds could be accomplished. We finally printed out three kinds of scaffolds by setting different filling rates: scaffold I with a filling rate of 90%, while scaffold II and scaffold III with filling rates of 50% and 20%, respectively. The detailed fabrication parameters of the three scaffolds are shown in Table 1.

### 2.4. Isolation and Culture of Bone Marrow Mesenchymal Stem Cells (BMSCs)

BMSCs of New Zealand white rabbits were cultured by the whole bone marrow adherent method. In brief, the rabbits were anesthetized with 1.5% pentobarbital (2 mL/kg) via ear veins, and the hair of the puncture site was completely shaved. After sterilization, 4-5 mL of bone marrow serum from iliac crest was extracted by a medullo-puncture needle and quickly transferred to the preheparinized centrifuge tube. The bone marrow serum was cultured in complete high-glucose DMEM (supplemented with 10% FBS, 100 U/mL penicillin, and 100 *μ*g/mL streptomycin). Half of the medium was exchanged every 3 days for three times and completely exchanged every 2-3 days afterwards. Then, the morphology and growth of the BMSCs were observed under the light microscope. Cells that reached 80-90% confluence were digested by 0.25% trypsin and passaged at 1 : 2 ratios. BMSCs from passage of 3rd to 5th were harvested for experiments.

### 2.5. Scanning Electron Microscope (SEM)

The microstructure and biocompatibility of the PLLA scaffolds with different pore sizes were evaluated by SEM (FEI Quanta 250, USA). Briefly, the 3D-printed PLLA scaffolds were seeded with BMSCs in a density of 1 × 10^5^ cells/mL after cell counting. Seven days later, the scaffold-cell composites were fixed with 2.5% glutaraldehyde, dehydrated in graded alcohols, replaced with isoamyl acetate, and dealt with critical point drying. After coating with gold by an ion sputtering coating machine (Quorum, UK), the scaffolds and the scaffold-cell composites were stuck on the observation platform with a double-sided adhesive tape and then observed under the SEM.

### 2.6. Mechanical Analysis

Mechanical properties of PLLA scaffolds were tested with the Instron Testing Machine (PA, USA). The data of stress-strain curve, compression modulus, and elastic modulus were recorded and analyzed.

### 2.7. Cell Counting Kit-8 (CCK-8) Assay

The PLLA scaffolds were irradiated with ultraviolet and then immersed in complete medium (10 mL/cm^2^) as the standard extract. 0.5x, 2x, and 4x extracts were prepared by the same method and placed in the 37°C, 5% CO_2_ incubator for 48 hours.

BMSCs were digested and seeded in 96-well plates (1 × 10^5^/mL, 100 *μ*L/well). After cell adherence, the original medium was replaced by different concentrations of extracts and cultured for 3 days. Cells in complete medium were recognized as negative control. Subsequently, 10 *μ*L of CCK-8 solution was added into each well and incubated at 37°C for 2 hours. Then, the microplate spectrophotometer (Tecan, USA) was applied to measure the optical density (OD) value of these samples at 450 nm. The relative growth ratio (RGR, RGR = (OD value of the samples/OD value of the negative control) × 100%) represents the toxicity of PLLA scaffolds.

The proliferation of BMSCs on three different scaffolds was also detected. In brief, BMSCs were cultured on scaffolds in 24-well plates (1 × 10^5^/mL, 500 *μ*L/well) for 1-5 days. The cells that seeded in the culture dishes were used as the control group. Next, 50 *μ*L of CCK-8 solution was added into each well at each time point and incubated at 37°C for another 2 hours. After the first step reaction, the reacted solution was transferred to 96-well plates and the absorbance was measured at 450 nm.

### 2.8. Bone Regeneration on Scaffolds In Vitro

1 × 10^6^ BMSCs were digested and seeded on the scaffolds with different pore sizes. After osteogenic differentiation induction for 7 days, the cells on the scaffolds were washed with phosphate buffer saline (PBS) and fixed with 4% paraformaldehyde, then stained with alkaline phosphatase (ALP) dye for 30 minutes and photographed. The ALP activity was also quantified by measuring the OD value of para-nitrophenol at 405 nm after cell lysis and normalized to the total protein measured by BCA. Three replicates of each scaffold were analyzed.

### 2.9. Construction of the In Vivo Bioreactor

The animal experiments were approved by Shanghai Jiao Tong University Animal Care and Use Committee and conducted in accordance with the “Guide for Care of Laboratory Animals” outlined by the National Ministry of Science.

**Figure 2:**
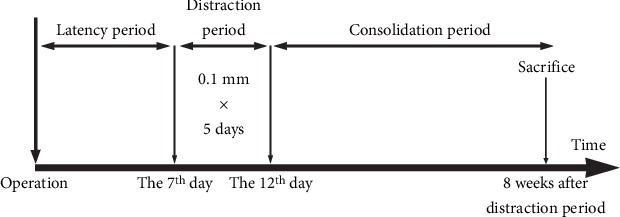
Process of PDO. PDO can be divided into three stages: they are the latency period, distraction period, and consolidation period. Seven days after surgery operation, it is the time for the latency period. Then, we adopted a rate of 1.0 mm/day for 5 days in the distraction period. Finally, the animals were sacrifice and the skull samples were collected after 8 weeks of the consolidation period.

**Figure 3:**
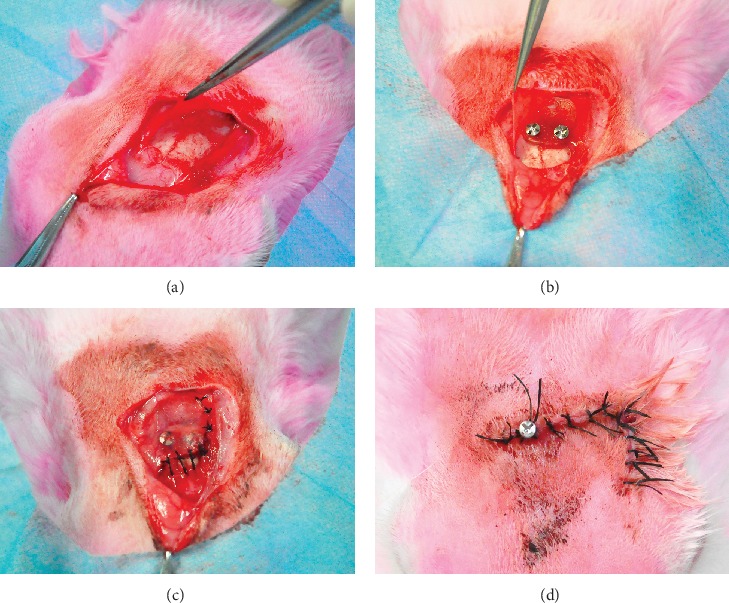
Construction of the *in vivo* bioreactor. (a) Separate the skin and periosteum on the rabbit skull. (b) The PLLA scaffold was fixed on the skull bone by two self-drilling screws under the periosteum. (c) Suture the periosteum *in situ* after covering the whole distraction scaffold. (d) Seven days later, a self-tapping screw was applied to uplift the PLLA scaffold to distract the periosteum.

The construction of the *in vivo* bioreactor process is shown in Figure 2. In brief, rabbits were anesthetized preoperatively with an intravenous administration of 1.5% pentobarbital (2 mL/kg) into the lateral ear veins. The forehead was shaved and disinfected with 3% iodophor followed by 75% ethyl alcohol. Next, 0.5 mL lidocaine (0.25%) was injected into the operation site before surgery. An L-shaped incision was conducted on the forehead. After separating the skin from the periosteum, we cut the periosteum with another L-shaped incision from the opposite site and carefully stripped it to fully expose the skull bone (Figure 3(a)). It is necessary to protect the adjacent superficial temporal vessels and the integrity of the periosteum. Then, the PLLA scaffold was inserted into the subperiosteal space and fixed on the cortical bone of the skull by two self-drilling screws (Figure 3(b)). After fully covering the whole material, the periosteum and the skin were sutured *in situ* (Figures 3(c) and 3(d)). After 7 days of latency period, we removed a part of the suture of the skin and made a 2 mm incision of the periosteum over the distraction hole of the scaffold. The self-tapping screw was inserted into the hole and rested on the skull external lamina, it can advance 0.5 mm by each 180° turn (Figure 3(d)). The distraction period lasts for 5 days at a rate of 1.0 mm/day. All animals received intramuscular injection of antibiotics for three days after surgery. During the consolidation period, attention should be paid to prevent screw loosening and wound infection. Finally, all rabbits were sacrificed with air embolism after 8 weeks. The sample of the skull and the distraction device were removed together and fixed in 4% paraformaldehyde.

### 2.10. Micro-CT Scanning

After fixation for 48 hours, the specimens were scanned by a high-resolution micro-CT imaging system (Scanco *μ*CT100, Swiss) with 20 *μ*m continuous increments at 90 kV and 200 *μ*A. PLLA materials were not developed during micro-CT scanning. After removing the noise of the scanned images through the method of median filter, we separate bone tissue from the high-density titanium nail by adjusting the maximum threshold to 70, which is the highest threshold of bone tissue. 100 scanning layers above the skull bone were reconstructed; then, Scanco *μ*CT100 evaluation software was applied to quantify the reconstructed area.

### 2.11. Histology Examination

The skull specimens (removing the distraction device) were decalcified in 12.5% ethylene diamine tetracetic acid (EDTA) solution for 2 months, rinsed under running water overnight, dehydrated in graded alcohols, and subsequently embedded in paraffin before histological staining. The embedded tissues were cut into thickness of 7 *μ*m along the axis of the distraction area. Hematoxylin and eosin (H&E) staining was conducted for the analysis of the newly formed bone. Immunohistochemical (IHC) staining was performed to detect the expression of osteocalcin (OCN) and CD31. Briefly, the deparaffinized sections were repaired with pepsin, blocked endogenous peroxidase activity with 3% H_2_O_2_, then incubated overnight with mouse monoclonal anti-OCN antibody (Abcam, ab13418) and anti-CD31 antibody (Abcam, ab9498) at 4°C. Negative control was treated with PBS. Afterwards, the sections were incubated with goat anti-mouse IgG-HRP (Maixin, China) at room temperature for 1 hour. Finally, the staining was visualized with diaminobenzidine (DAB) and counterstained with hematoxylin. The slices were observed by a microscope (Zeiss, Germany). Image-Pro Plus system was applied to conduct quantitative analysis.

### 2.12. Statistical Analysis

All experiments were repeated about three times, and the statistical results were expressed as mean ± standard deviation (SD). Student's *t*-test was used to analyze the data between two groups, while one-way analysis of variance (ANOVA) was performed to analyze the statistical differences for multiple comparisons. *P* < 0.05 was considered statistically significant, and *P* < 0.01 and *P* < 0.001 were recognized as highly statistically significant.

## 3. Results

### 3.1. Microstructure and Biocompatibility of PLLA Scaffolds with Different Pore Sizes

**Figure 4:**
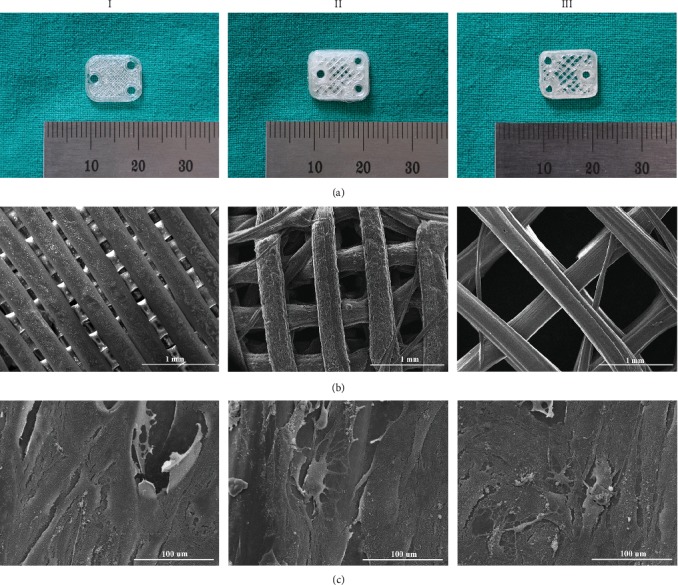
Microstructure and biocompatibility of PLLA scaffolds with different pore sizes. (a) Optical images of PLLA scaffolds I, II, and III. (b) SEM showed the interconnecting microchannels of scaffolds. (c) PLLA scaffolds with different pore sizes displayed good biocompatibility with BMSCs.

We designed a lamelliform structure (12 mm × 10 mm × 1 mm) with three 2 mm diameter holes at proper position (Figures 1(d) and 1(e)). As shown by the SEM, the PLLA scaffolds with different pore sizes showed a pattern of interlaced porous channels and good biocompatibility (Figure 4). The BMSCs are fibrous in shape and extended pseudopodia to adhere to the surface of scaffolds.

### 3.2. Influence of Filling Rates on Scaffold Properties

**Figure 5:**
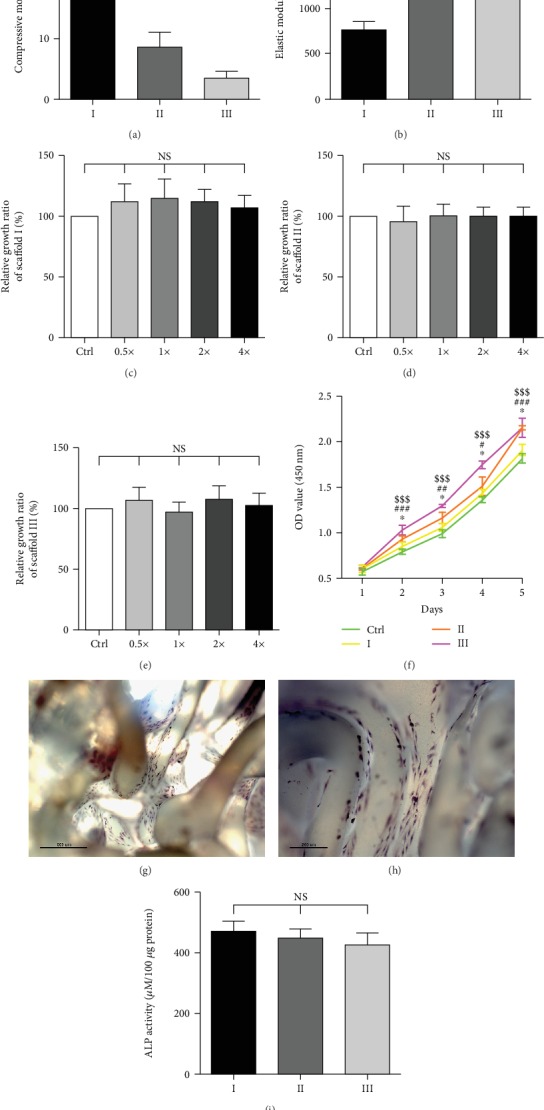
Influence of filling rates on scaffold properties. (a) Compressive modulus of PLLA scaffolds (*n* = 5, ^∗∗^*P* < 0.01, ^∗∗∗^*P* < 0.001). (b) Elastic modulus of different PLLA scaffolds (*n* = 5, ^∗∗∗^*P* < 0.001). (c) The influence of different concentrations of extracts from scaffold I on BMSC growth (*n* = 5, NS = not significant; Ctrl = control group). (d) The influence of different concentrations of extracts from scaffold II on BMSC growth (*n* = 5, NS = not significant; Ctrl = control group). (e) The influence of different concentrations of extracts from scaffold III on BMSC growth (*n* = 5, NS = not significant; Ctrl = control group). (f) The proliferation curves of BMSCs on scaffolds I, II, and III for 1-5 days (*n* = 5, ^∗^*P* < 0.05 vs. scaffold I, ^#^*P* < 0.05 vs. scaffold II, ^##^*P* < 0.01 vs. scaffold II, ^###^*P* < 0.001 vs. scaffold II, ^$$$^*P* < 0.001 vs. scaffold III, Ctrl = control group). (g) Alkaline phosphatase (ALP) staining of BMSCs on scaffolds after osteogenic differentiation for 7 days (40x) (scale bar = 500 *μ*m). (h) ALP staining of BMSCs on scaffolds after osteogenic differentiation for 7 days (100x) (scale bar = 200 *μ*m). (i) ALP activity of BMSCs on scaffolds after osteogenic differentiation for 7 days (NS = not significant).

3D printing technology could meet the requirement of printing out scaffolds with different filling rates. With the decreasing filling rates of the scaffolds, the porosity was gradually increased. The mechanical properties of PLLA scaffolds, such as compressive modulus and elastic modulus, gradually decreased with the decreasing filling rates (Figures 5(a) and 5(b)). However, there was no fracture in all three groups at the process of animal experiments.

We then conducted CCK-8 assays to measure the toxicity of PLLA scaffolds. The results showed that different concentrations of extracts had no significant influence on the cell growth relative to the corresponding negative control group, and there was no significant difference between different concentrations of extract (Figures 5(c)–5(e)). It can be concluded that PLLA is nontoxic and suitable for *in vivo* study, which is consistent with other literature reports [30–32].

The proliferation of BMSCs was observed for 1-5 days. The results of proliferation curves showed that BMSCs exhibited good viability and compatibility on three different scaffolds. Cells proliferated faster on scaffolds than in the control group; moreover, cells proliferated more rapidly on scaffolds with larger pore sizes than with smaller pore sizes (Figure 5(f)).

ALP staining could evaluate the effect of the bone regeneration *in vitro*. Staining at the surface of the PLLA scaffolds indicated that BMSCs could differentiate into osteoblasts after 7 days of osteogenic induction (Figures 5(g) and 5(h)). However, no significant difference of ALP activity was observed in the three experimental groups (Figure 5(i)).

### 3.3. Evaluation of the Regenerated Bone by Micro-CT Scanning

**Figure 6:**
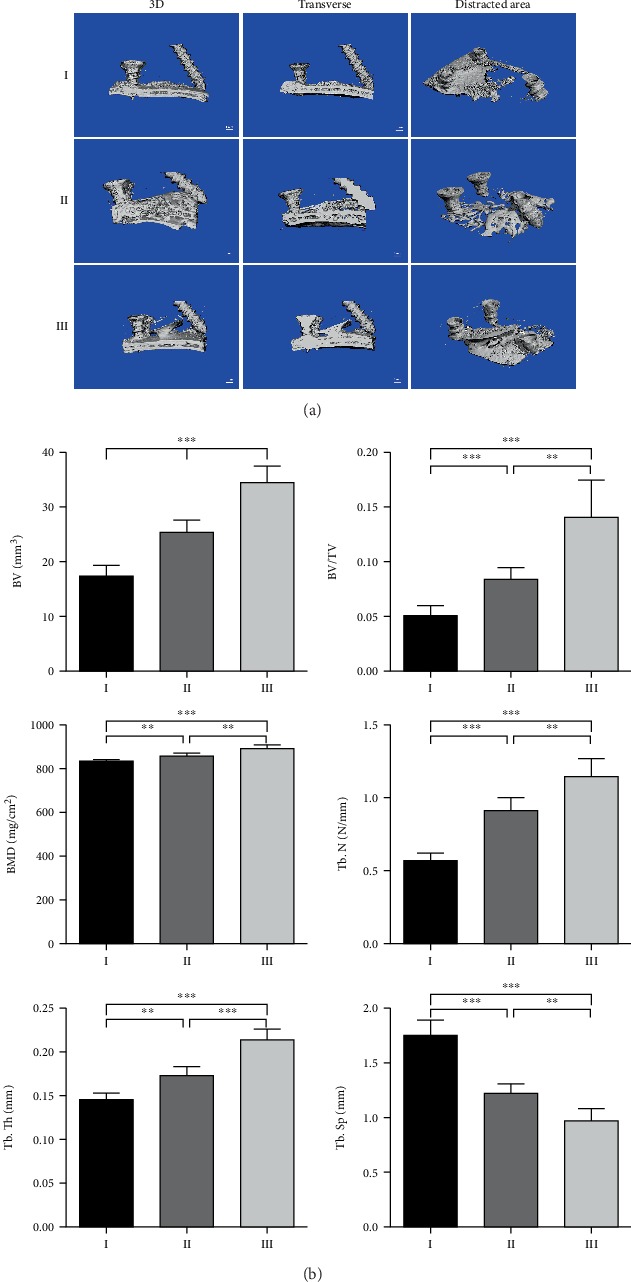
Evaluation of regenerated bone by micro-CT scanning. (a) Representative micro-CT scanning images, 100 scanning layers above the skull of the distracted area were reconstructed and analyzed. (b) The quantitative results of the bone volume (BV), bone volume/tissue volume (BV/TV), bone mineral density (BMD), trabecular number (Tb. N), trabecular thickness (Tb. Th), and trabecular separation (Tb. Sp). (*n* = 6, ^∗∗^*P* < 0.01, ^∗∗∗^*P* < 0.001).

As shown in Figure 6(a), there was new bone tissue formed in the gap between the skull and PLLA scaffold after 8 weeks of consolidation in all three groups. We could distinguish the cranial cortex from the newly formed trabeculas from the lateral view. The newly formed bone in the distracted area was reconstructed and quantified. The results showed that bone volume (BV), bone volume/tissue volume (BV/TV), and bone mineral density (BMD) were gradually increased with the increasing pore sizes of scaffolds. Moreover, the trabecular number (Tb. N) and trabecular thickness (Tb. Th) in groups with scaffolds of larger pore sizes were higher than those of smaller pore sizes, while the trabecular separation (Tb. Sp) showed the opposite tendency (Figure 6(b)).

### 3.4. Histological Examination of New Bone Formation

**Figure 7:**
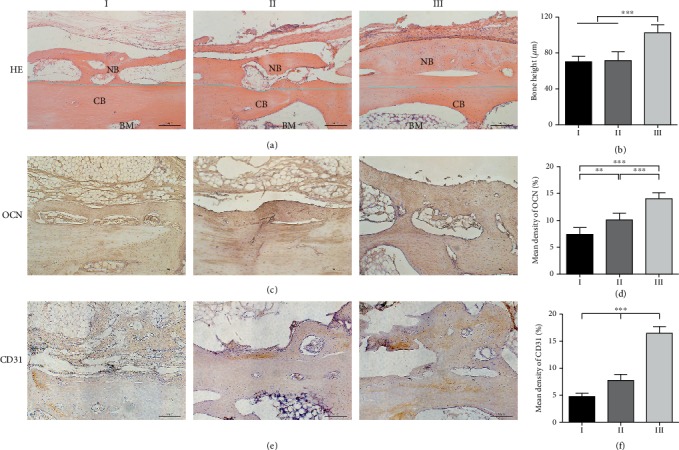
Histology examinations of the regenerated bone. (a) Hematoxylin and eosin (H&E) staining of specimens (NB: new bone; CB: cortical bone; BM: bone marrow. The blue line represents the boundary between the NB and the CB). (b) Bone height was measured by H&E staining (*n* = 3, ^∗∗∗^*P* < 0.001). (c) Osteocalcin (OCN) expression by immunohistochemistry (IHC) staining. (d) The mean density of OCN (%) was quantified. (*n* = 3, ^∗∗^*P* < 0.005, and ^∗∗∗^*P* < 0.001). (e) CD31 expression by IHC staining. (f) The mean density of CD31 (%) was quantified (*n* = 3, ^∗∗^*P* < 0.005, ^∗∗∗^*P* < 0.001).

H&E staining was used to assess the new bone formation. The newly formed bone existed at the space between the bone cortex and the distraction device and mostly deposited on the surface of the skull. With the increasing pore sizes of the scaffolds, the fibrous tissue was relatively reduced (Figure 7(a)), but the bone height was gradually increased (Figure 7(b)).

OCN and CD31 can be used as indicators to evaluate osteogenesis and angiogenesis. As shown in Figure 7(c), both of the cortical bone of the skull and the newly formed bone positively expressed OCN. With the increasing pore sizes of PLLA scaffolds, the boundary between the newly formed bone and the skull surface became more and more blurred. Quantitative results showed that the positive expression of OCN was increased with the increasing pore sizes of scaffolds (Figure 7(d)). CD31 was mainly expressed in the newborn tissue and displayed the same tendency with OCN (Figures 7(e) and 7(f)).

## 4. Discussion

To date, the PDO technique has been applied to the treatment of atrophic alveolar, cleft palate, and other congenital disease, although the clinical reports are relatively scarce [33]. There are many factors that affect this technology. In order to improve the effect of PDO, this study intended to utilize 3D printing technology to design the structure of the distractor. We printed out three kinds of PLLA scaffolds with different filling rates and then applied these scaffolds to distract the skull periosteum of rabbits for regenerating new bone tissue. It suggested that scaffolds with the larger pore sizes showed a better result of the regenerated bone. This phenomenon might be attributed to the reason that a large pore size is more conducive to the exchange of cells and cytokines between the periosteum and cortical bone; however, we should extend the consolidation period in our future study.

3D printing technology is advanced in meeting the requirements of scaffold properties, such as shapes, structures, sizes, and porosities [34, 35]. With the benefit of CAD/CAM system, we can print out customized scaffolds with precise architecture and obtain different pore sizes by regulating the filling rates. The filling rate is an important printing parameter of 3D printer, which determines how much material should be filled in the printed item, that is, the degree of how solid and hollow is the scaffold. Generally, it is set as a percentage, 0% is completely hollow, and 100% is completely solid. The higher the percentage of filling, the longer the printing time and the more materials are used. We can assume that the filling rate is inversely proportional to the porosity. The larger is the filling rate, the smaller is the porosity. It should be noted that the change of filling rates not only decides the porosity but also does change the surface area of scaffold. Different porosity and surface area might affect cell adhesion, proliferation, and differentiation; this may explain why there is no difference in the activity of ALP *in vitro*. In fact, it is difficult to control a single factor when studying their influence on the PDO. As shown in our study, the pore sizes of the scaffolds were gradually increased with the decrease of the filling rates, and the mechanical strength of the material decreased either. The scaffolds require different pore sizes and sufficient mechanical strength simultaneously; thus, it is significant to explore appropriate parameters in the future so that the printed scaffolds can not only have customized structures but also achieve adequate strength.

As for the design of distraction device, we adopted the simple distractor to form a wedge gap between the periosteum and the cortical bone, which not only prevented the invasion of the surrounding tissues but also provided a space for bone formation. On the one hand, this kind of device could decrease tissue injury compared with the traditional U-shaped distractor; on the other hand, it could achieve accurate distraction by uplifting 0.5 mm with each 180° turn of the distraction screw. Initially, titanium alloy and stainless steel were used for constructing the distraction device. Researchers then began to use biodegradable materials to fabricate scaffolds, including hydrogel, beta-tricalcium phosphate (*β*-TCP), PLLA/HA, poly-DL-lactide (PDLLA), and polyglycolic acid (PGA) [18, 36–38]. A recent study proved that there was no difference in the formation of the new bone between the degradable materials and metal materials for the PDO [38]. However, biodegradable materials are more suitable for the construction of tissue engineering scaffolds, because it can reduce the polymer residues in the repair site [39]. In our study, we applied the biodegradable polymer PLLA to manufacture scaffolds, and the results from the SEM and CCK-8 assays demonstrated that PLLA had good biocompatibility. Nevertheless, the ideal scaffold material for bone tissue engineering needs good biocompatibility, mechanical strength, and osteoinductivity at the same time. Natural materials possess good biocompatibility and degradability, but the mechanical strength is relatively low. Therefore, the composite material formed by natural polymer and synthetic polymer is very promising in the future. The addition of synthetic polymer could improve the mechanical strength of the composite material, so that the overall biological properties of the material are more suitable for the formation of the new bone [39]. The limitation of PLLA lies in the osteoinductivity; thus, in our future study, we can combine the PLLA with some other osteoinductive materials, such as *β*-TCP and HA. We should also focus on exploiting the 3D printing of other functional materials for a tissue-engineered bone in the future. The technology of tissue engineering provides an alternative method to fabricate native tissues by creating an artificial environment to facilitate the growth of cells. It is aimed at constructing biological substitutes for *in vivo* transplantation [40, 41]. Driven by the advancement of biomaterials and fabrication techniques, tissue engineering could satisfy the requirement of constructing 3D scaffolds with controlled structure and porosity for providing a supportive matrix for the growth of bone tissue [39, 42].

Periosteum tissue covers the majority of bone surface except for the joints. In our study, we chose the skull periosteum for distraction. Researchers initially implanted the distraction devices under the periosteum of mandible bone, but the operation is relatively difficult, and the device would fall off because of the chewing activity [13, 19]. Stevens et al. created a space between the tibia and the tibial periosteum of the rabbit to obtain the new bone and successfully repaired the contralateral defect [18]. This method could not regenerate enough bone tissue for the treatment of large bone defects because of the limited space. Then, a number of researchers have begun to study the PDO under the periosteum of the skull [37, 38, 43–51]. The skull is flatter and more suitable for the placement of distraction devices; moreover, the periosteum of the skull is more robust and easier to separate compared with other positions. Therefore, the calvarial periosteum is suitable for PDO research. The periosteum acts as a barrier to prevent the invasion of soft tissue, so it is critically important to maintain the integrity of periosteum in the process of animal experiments. In addition, the distraction screw should be positioned far from the incision of the periosteum so as to avoid dehiscence.

Osteogenesis and angiogenesis are two interconnected processes, and the survival of new bone tissue depends on the regeneration of new vessels [52]. The application of a *in vivo* bioreactor can significantly improve the vascularization of the tissue-engineered bone [53–55]. As the main source of blood supply for bone tissue, the periosteum can be used to construct the *in vivo* bioreactor and is beneficial for angiogenesis [15, 16, 52]. Our study showed that the vascularization of the new bone increased with the increasing pore sizes of the distractors, which is closely related to the role of the *in vivo* bioreactor. In previous studies, researchers have explored other ways to promote the osteogenesis and angiogenesis of PDO, including cortical bone perforation and BMSC injection [56–58]. MSCs, as a kind of stem cells with multiple differentiated potential, are recognized as the most ideal seed cells for tissue engineering because of their wide sources and stable phenotypes [59, 60]. In addition, it has been well documented that MSCs can produce enough vascular endothelial growth factor (VEGF) to promote angiogenesis [61]. Perforating on bone surface could facilitate the increase of bleeding and release of BMSCs, which is also benefit for angiogenesis as well as osteogenesis. Therefore, BMSC administration combined with cortical bone perforation can overcome the problem of insufficient osteogenic cells, but the specific drilling method and injection frequency still need to be explored.

## 5. Conclusions

In conclusion, 3D-printed PLLA scaffolds with a customized structure and different pore sizes have been successfully constructed. These scaffolds exhibited excellent bioactivity and biocompatibility *in vitro*, while the mechanical properties were gradually decreased with the increasing pore sizes. We tried to investigate the influence of pore sizes on the distractor on the PDO; the results showed that distractors with larger pore sizes were more favorable for new bone regeneration. We hope to combine the technologies of 3D printing, PDO, and *in vivo* bioreactor to fabricate scaffolds for regenerating adequate bone tissue in the future.

## Figures and Tables

**Table 1:** Printing parameters.

Scaffold	Filling ratio (%)	Layer height (mm)	Number of layers	Number of shells	Speed (mm/s)	Temperature (°C)
I	90	0.2	5	2	60	180
II	50	0.25	4	2	60	180
III	20	0.25	4	2	80	180

## Data Availability

The data used to support the findings of this study are available from the corresponding author upon request.
